# Guselkumab Every 4 Weeks as a Real-World Therapeutic Option for Psoriatic Arthritis Patients at High Risk of Joint Damage

**DOI:** 10.3390/clinpract16080146

**Published:** 2026-08-07

**Authors:** Nicoletta Bernardini, Giorgio Marotta, Nevena Skroza, Dario Graceffa, Maria Consiglia Bragazzi, Lucia Finistauri Guacci, Ersilia Tolino, Francesca Paola Sasso, Umberto Gallo, Antonio Giovanni Richetta, Annunziata Dattola

**Affiliations:** 1 Dermatology Unit “Daniele Innocenzi”, Department of Medical-Surgical Sciences and Biotechnologies, La Sapienza University of Rome, Polo Pontino, 04100 Latina, Italy; nicoletta.bernardini@libero.it (N.B.); nevena.skroza@uniroma1.it (N.S.); ersiliatolino@gmail.com (E.T.); 2 Unit of Dermatology, Department of Medical and Cardiovascular Sciences, La Sapienza University of Rome, 00161 Rome, Italy; luciafinistauri1996@gmail.com (L.F.G.); francescapaolasasso@gmail.com (F.P.S.); umbertogallo2@gmail.com (U.G.); antonio.richetta@uniroma1.it (A.G.R.); nancydattola@gmail.com (A.D.); 3 Department of Clinical Dermatology, San Gallicano Dermatological Institute, IRCCS, 00144 Rome, Italy; dario.graceffa@ifo.it; 4 Gastroenterology Unit, Department of Medical-Surgical Sciences and Biotechnologies, La Sapienza University of Rome, Polo Pontino, 04100 Latina, Italy; mariaconsiglia.braga@uniroma1.it

**Keywords:** psoriatic arthritis, guselkumab, IL-23 inhibitor, joint damage progression, DAPSA, real-world evidence, every-4-week dosing

## Abstract

**Background:** Guselkumab, a monoclonal antibody selectively targeting the p19 subunit of interleukin-23, is typically administered with an induction phase at weeks 0 and 4 followed by maintenance dosing every 8 weeks for the treatment of psoriatic arthritis (PsA). In Europe, a four-week regimen (Q4W) has recently been approved for patients at high risk of joint damage progression; however, real-world evidence on this intensified schedule remains limited. This study aimed to evaluate the effectiveness and safety of guselkumab Q4W in routine clinical practice. **Methods:** This retrospective observational study included 18 PsA patients at high risk of structural damage treated with guselkumab Q4W at two psoriasis referral centers in the Lazio region of Italy. Baseline and 24-week clinical data were collected from medical records. Outcome measures included Disease Activity Index for Psoriatic Arthritis (DAPSA), Minimal Disease Activity (MDA), pain Visual Analog Scale (VAS), Dermatology Life Quality Index (DLQI), and Psoriasis Area and Severity Index (PASI). **Results:** After 24 weeks, mean DAPSA decreased from 34.3 ± 7.6 to 3.7 ± 3.0; 55.6% of patients achieved DAPSA remission and 77.8% achieved MDA. Significant improvements were observed in pain VAS, DLQI, and PASI scores. All patients achieved PASI 75, while 83.3% achieved PASI 90 and PASI 100. Treatment was generally well tolerated, with no adverse events or new safety signals documented during follow-up. **Conclusions:** In our study, guselkumab Q4W showed marked multidomain effectiveness and a favorable safety profile, supporting its use in PsA patients at high risk of joint damage progression.

## 1. Introduction

Psoriatic arthritis (PsA) is a chronic inflammatory disease affecting approximately 20–30% of patients with psoriasis, with a global prevalence estimated at 112 per 100,000 adults and a rising incidence over the past two decades [1,2]. It is characterized by heterogeneous clinical manifestations, including peripheral joint inflammation, enthesitis, and inflammatory axial involvement [3].

The disease burden can be significant, as some patients may develop disabling forms of arthritis characterized by multiple bone erosions with consequent severe functional impairment and reduced quality of life [4]. PsA may also be associated with extra-articular manifestations including uveitis, inflammatory bowel disease, cardiometabolic and psychiatric disorders [5].

The diagnosis of PsA is based on the recognition of clinical and imaging characteristics, as no specific biomarkers are currently available [6,7]. Early diagnosis is crucial: a diagnostic delay as short as six months has been associated with the development of irreversible joint damage and lower response to therapy [8]. Conversely, early intervention with disease-modifying antirheumatic drugs (DMARDs) can significantly improve both clinical outcomes and radiographic damage, supporting the concept of a “window of opportunity” for therapeutic intervention [9].

The pathogenesis of PsA involves complex interactions between genetic predisposition and environmental triggers, resulting in an inflammatory response mediated by dendritic cells, T cells, and cytokines such as tumor necrosis factor alpha (TNF-α) and the IL-23/IL-17 pathway. IL-23 is recognized as a key driver of joint inflammation through the activation and expansion of Th17 lymphocytes and subsequent release of proinflammatory cytokines including IL-17, IL-22, and TNF-α [10].

The introduction of biologic therapies targeting cytokines, such as anti-TNF-α, anti-IL-17, anti-IL-12/23 and anti-IL-23 agents, has revolutionized the management of both psoriasis and PsA [11].

The updated 2023 EULAR recommendations and the 2021 GRAPPA treatment recommendations advocate a treat-to-target, individualized approach to PsA management, with escalation to a biologic DMARD following an inadequate response to conventional therapy. For peripheral joint disease, no clear preference is given among TNF, IL-17, and IL-23 inhibitors, whereas the presence of extra-musculoskeletal manifestations (particularly skin disease, uveitis, and inflammatory bowel disease), comorbidities, and safety considerations should guide treatment selection. Janus kinase inhibitors are also recommended after inadequate response to csDMARDs; however, EULAR generally reserves their use for patients with an inadequate response to a biologic DMARD or when a biologic is not appropriate, whereas GRAPPA includes them among the recommended advanced treatment options after csDMARD failure [12,13,14].

A recent study evaluated the 5-year risk of developing PsA in a cohort of patients with psoriasis treated with IL-23 inhibitors (guselkumab, risankizumab, tildrakizumab) or IL-17 inhibitors (ixekizumab, secukinumab), showing that patients receiving IL-23 inhibitors had a lower risk of PsA development as compared with the IL-17 inhibitor group [15]. Furthermore, emerging real-world evidence suggests that IL-23 inhibitors may effectively control early musculoskeletal manifestations in patients with psoriasis and could potentially delay progression to overt PsA, although prospective validation is still needed [16].

Guselkumab selectively targets the p19 subunit of IL-23, offering a distinct mechanism of action. Guselkumab has proven to have sustained effectiveness and safety up to two years in patients with psoriasis and active PsA [17]. Regarding the rheumatologic domain, recent data demonstrated that guselkumab achieved significantly higher rates of clinical improvement and inhibition of structural damage progression compared with placebo, with no safety issues and consistent multidomain efficacy [18,19].

Guselkumab is approved for the treatment of moderate-to-severe plaque psoriasis, active PsA, and moderately to severely active ulcerative colitis and Crohn’s disease. The main indications and recommended dosages are summarized in Table 1 [20].

In PsA, guselkumab is typically administered subcutaneously every eight weeks as maintenance therapy. However, in Europe, a four-week dosing regimen is also approved for patients at high risk of joint damage progression [20].

Although several real-world studies have confirmed the effectiveness and persistence of guselkumab administered according to the standard every-8-week regimen, evidence on the intensified every-4-week schedule remains scarce [21].

The aim of the present study was to evaluate the efficacy of guselkumab administered subcutaneously every four weeks in patients with PsA in a real-world setting, assessing both the skin and joint domains.

## 2. Materials and Methods

This retrospective observational study was conducted across two psoriasis referral centers in the Lazio region of Italy (Policlinico Umberto I and Polo Pontino). Patients initiated guselkumab between April and July 2025, and all completed the Week-24 assessment by December 2025. Eighteen patients were included; all were initially managed for chronic plaque psoriasis and, during routine outpatient follow-up, developed inflammatory articular symptoms consistent with psoriatic arthritis. Owing to inadequate response to previous conventional therapies and the presence of a high risk of disease progression and joint damage, treatment escalation was deemed necessary. In agreement with the consulting rheumatologist, all patients were initiated on guselkumab administered every four weeks.

The diagnosis of PsA was established according to the Classification Criteria for Psoriatic Arthritis (CASPAR); all 18 patients included in the study fulfilled the CASPAR criteria [6]. No patients were lost to follow-up or discontinued guselkumab before Week 24; all 18 patients completed the 24-week observation period. Based on review of medical records, during the 24-week follow-up, no patients received concomitant csDMARDs, systemic corticosteroids, or topical antipsoriatic therapies. NSAIDs could be used on an as-needed basis for symptom control. Inadequate response to previous therapy was defined, in line with treat-to-target recommendations for PsA, as failure to achieve Minimal Disease Activity (MDA) despite an adequate trial of conventional and/or biologic therapy, together with persistently elevated composite (DAPSA) and skin (PASI) disease activity, corroborated by clinical/rheumatological judgment regarding the risk of structural progression. The specific conventional and biologic agents previously used by each patient are summarized in Table 2; in all cases, the reason for switching to guselkumab was inadequate response as defined above.

Patients were classified as being at high risk of joint damage progression on the basis of individualized clinical and rheumatological judgment, in accordance with the product label indication for Q4W dosing, which does not specify a standardized operational definition [20]. This judgment took into account the overall disease profile, including polyarticular involvement, persistently high disease activity, elevated CRP, and previous failure of conventional and/or biologic therapies; these features were not required to be simultaneously present in every patient.

Dermatologic and rheumatologic assessments routinely performed in clinical practice at baseline (T0) and after 24 weeks of treatment were retrospectively collected from medical records.

Disease activity was assessed using the Disease Activity index for Psoriatic Arthritis (DAPSA) and Minimal Disease Activity (MDA).

DAPSA was calculated as the numerical sum of tender joint count (TJC, 68 joints), swollen joint count (SJC, 66 joints), patient global assessment of disease activity (VAS, 0–10 cm), patient pain assessment (VAS, 0–10 cm) and C-reactive protein (CRP, mg/dL).

MDA was defined as fulfillment of at least 5 out of the following 7 criteria: TJC ≤ 1/68, SJC ≤ 1/66, PASI ≤ 1 or BSA ≤ 3, Pain VAS (0–10 cm) ≤ 1.5 cm, patient global assessment of disease activity (VAS, 0–10 cm) ≤ 2.0 cm, Health Assessment Questionnaire–Disability Index (HAQ-DI) ≤ 0.5 and Leeds Enthesitis Index (LEI) ≤ 1.

In addition to these composite scores, pain intensity, quality of life, and skin involvement were assessed using the Visual Analog Scale (VAS), the Dermatology Life Quality Index (DLQI) and the Psoriasis Area and Severity Index (PASI), respectively. In addition to the changes in mean Psoriasis Area and Severity Index (PASI) score, we also evaluated the proportion of patients achieving PASI 75, PASI 90, and PASI 100 responses at Week 24.

Sixteen patients received guselkumab 100 mg every four weeks, while two patients with concomitant ulcerative colitis (UC) received guselkumab 200 mg every four weeks, according to disease severity and prescribing information, following consultation with gastroenterology specialists.

### 2.1. Statistical Analysis

Descriptive statistics were used to summarize demographic and clinical characteristics. Continuous variables were reported as mean ± standard deviation (SD), while categorical variables were expressed as absolute numbers and percentages.

Normality of the distribution of changes (Week 0 to Week 24) in continuous outcomes, including DAPSA, pain Visual Analog Scale (VAS), Dermatology Life Quality Index (DLQI), and Psoriasis Area and Severity Index (PASI), was assessed using the Shapiro–Wilk test. For DAPSA, whose changes were normally distributed (*p* = 0.31), a paired *t*-test was used. For pain VAS, DLQI, and PASI, whose changes departed significantly from normality (*p* = 0.005, *p* = 0.013, and *p* = 0.038, respectively), the non-parametric Wilcoxon signed-rank test was used instead. Mean changes from baseline were calculated together with their corresponding 95% confidence intervals (CI) for the normally distributed variable (DAPSA); median changes are reported for the non-normally distributed variables (pain VAS, DLQI, PASI).

Categorical outcomes, including achievement of Minimal Disease Activity (MDA) and PASI 75/90/100 responses at Week 24, were summarized as proportions.

Statistical analyses were performed using GraphPad Prism version 10.6.1 (GraphPad Software, San Diego, CA, USA). A two-sided *p*-value < 0.05 was considered statistically significant.

### 2.2. Patient Baseline Characteristics

The cohort consisted of 18 patients with a confirmed diagnosis of PsA, including 8 males (44.4%) and 10 females (55.6%). The mean age was 54.72 years (SD = 12.64), and the mean disease duration was 12.18 years (SD = 8.43). Seven patients (38.9%) reported a positive family history of psoriasis.

Regarding the predominant articular phenotype, 13 patients (72.2%) had predominantly peripheral involvement, whereas 5 patients (27.8%) had predominantly axial involvement. Patients were categorized according to the predominant articular manifestation documented in the medical records; these descriptive categories reflected the dominant clinical phenotype and did not exclude the coexistence of peripheral and axial symptoms. All patients, including those with axial-predominant disease, had measurable peripheral joint involvement; therefore, DAPSA was calculated across the entire cohort. Dactylitis was reported in 7 patients (38.9%), and enthesitis in 11 (61.1%). Nail psoriasis was documented in 10 individuals (55.6%).

All patients experienced previous treatment failure of conventional systemic DMARDs and all had been exposed to at least one prior biologic therapy, specifically adalimumab. Notably, 9 patients (50.0%) failed multiple biologic agents before the start of guselkumab, including secukinumab in 5 cases (27.8%), infliximab in 2 (11.1%), and ixekizumab in 2 (11.1%).

Cardiometabolic comorbidities were identified in 15 patients (83.3%). Importantly, concomitant ulcerative colitis (UC) was observed in 2 individuals (11.1%). A detailed summary of the demographic and clinical characteristics of the study cohort is provided in Table 2.

## 3. Results

After 24 weeks of treatment with guselkumab, improvements were observed across all clinical and patient-reported outcomes.

**Figure 1:**
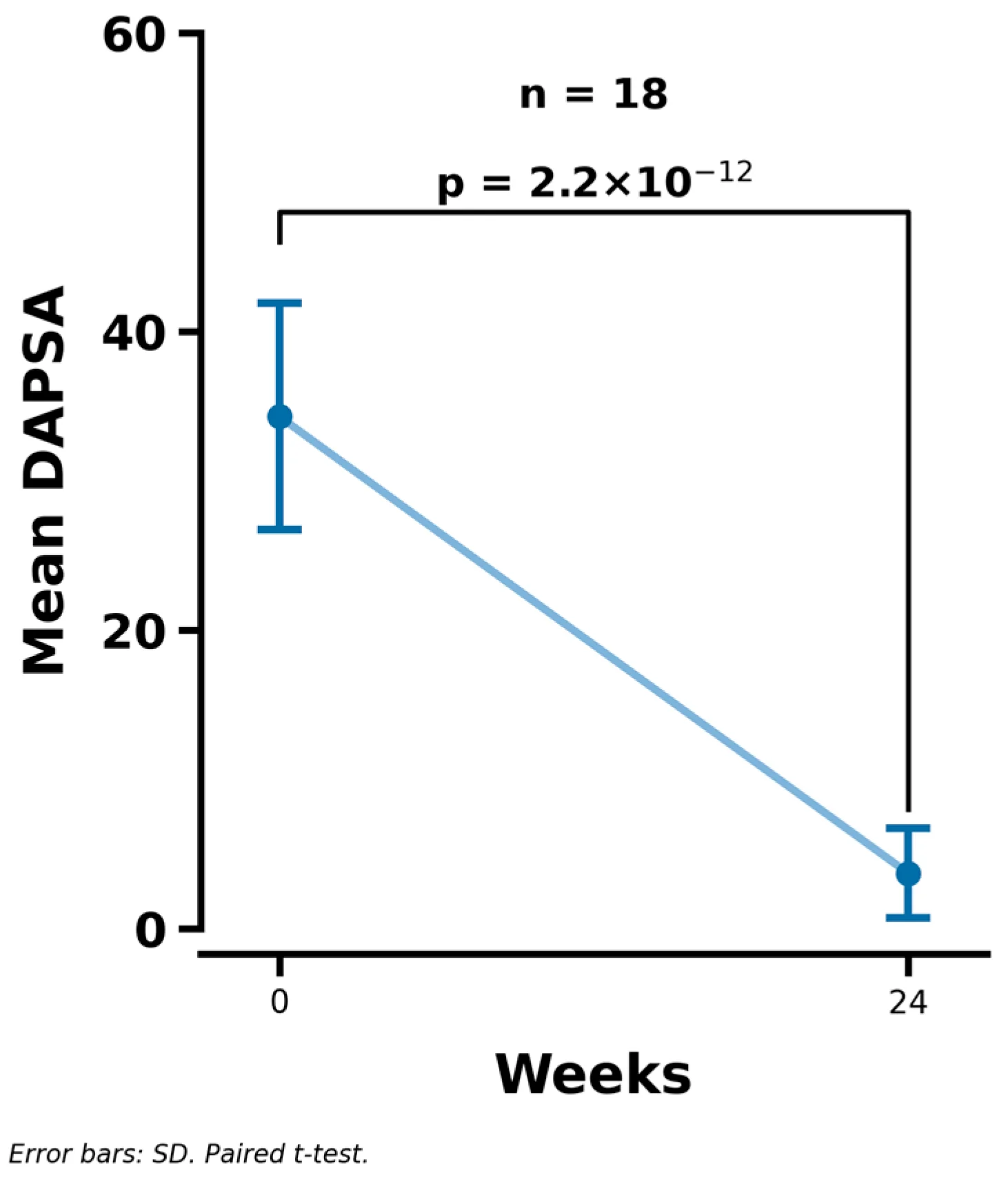
Mean Disease Activity in Psoriatic Arthritis (DAPSA) scores at baseline and at Week 24. Mean DAPSA declined from 34.3 ± 7.6 at baseline to 3.7 ± 3.0 at Week 24, indicating a profound reduction in overall psoriatic arthritis disease activity. *n* = 18. Error bars represent standard deviation (SD); *p* = 2.2 × 10^−12^, paired *t*-test.

After 24 weeks of treatment, the mean DAPSA decreased from 34.3 ± 7.6 to 3.7 ± 3.0, corresponding to a mean change of −30.66 points (95% CI −34.38 to −26.94; *p* = 2.2 × 10^−12^), indicating a marked reduction in disease activity (Figure 1). At Week 24, 10 out of 18 patients (55.6%) achieved DAPSA remission (DAPSA ≤ 4), and the remaining 8 patients (44.4%) achieved low disease activity (DAPSA ≤ 14), with no patients in moderate or high disease activity.

**Figure 2:**
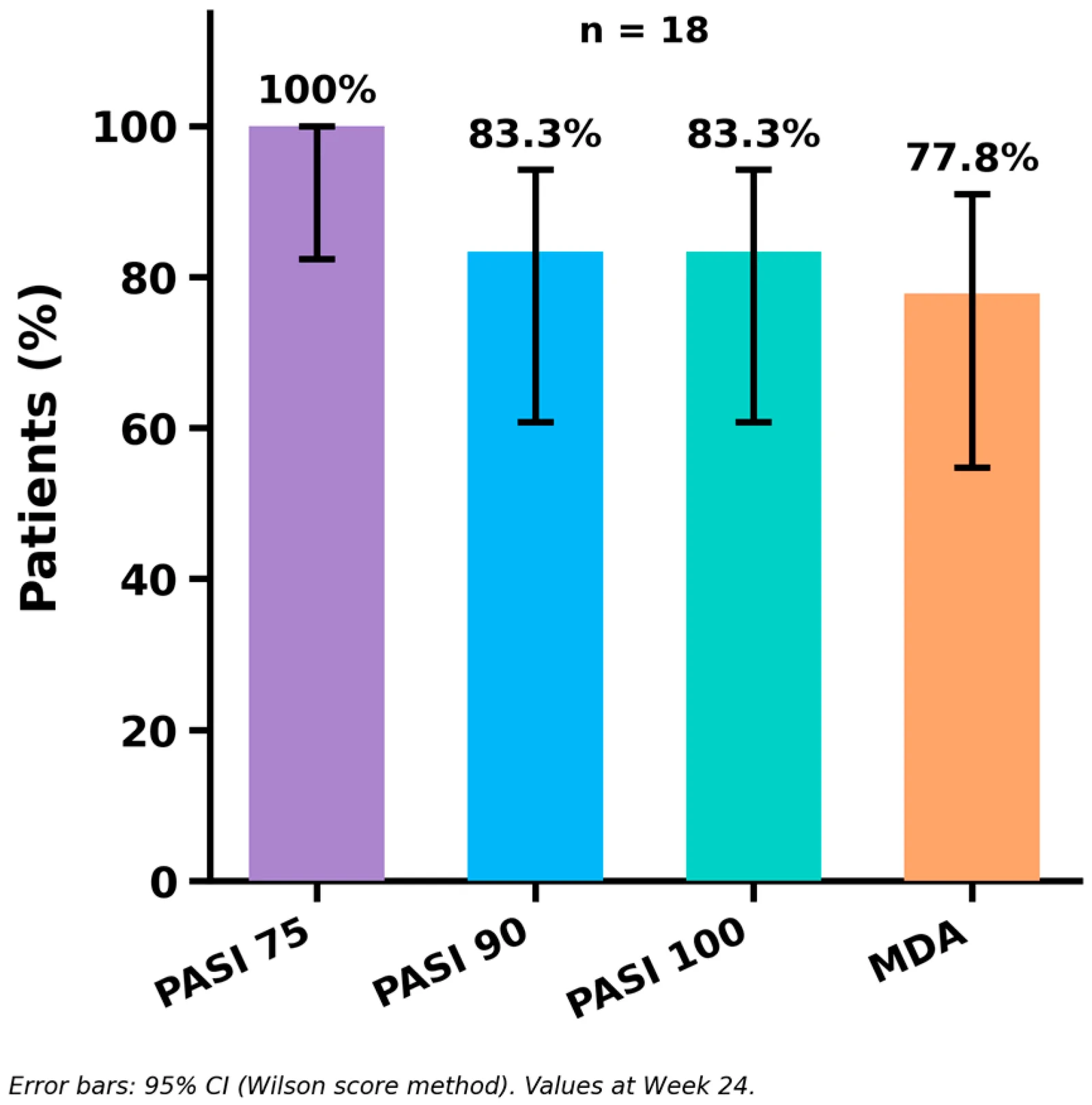
Proportion of patients achieving PASI 75, PASI 90, PASI 100, and Minimal Disease Activity (MDA) at Week 24. All patients (100%) reached PASI 75, while 83.3% achieved both PASI 90 and complete skin clearance (PASI 100). Additionally, 77.8% of patients met the criteria for MDA, demonstrating substantial improvements in both dermatologic and rheumatologic domains. *n* = 18. Error bars represent 95% confidence intervals (Wilson score method).

A total of 14 patients (77.8%) achieved Minimal Disease Activity (MDA) according to the predefined criteria (Figure 2).

Mean pain VAS significantly decreased from 5.89 ± 1.28 cm at baseline to 0.56 ± 0.62 cm at Week 24 (median change of −5.0 cm; Wilcoxon signed-rank test, *p* = 0.0002), reflecting a significant improvement in pain perception.

Similarly, mean DLQI significantly improved from 15.94 ± 7.41 at baseline to 1.50 ± 1.50 at Week 24 (median change of −11.5; Wilcoxon signed-rank test, *p* = 0.0002), indicating a marked enhancement in patient-reported quality of life.

**Figure 3:**
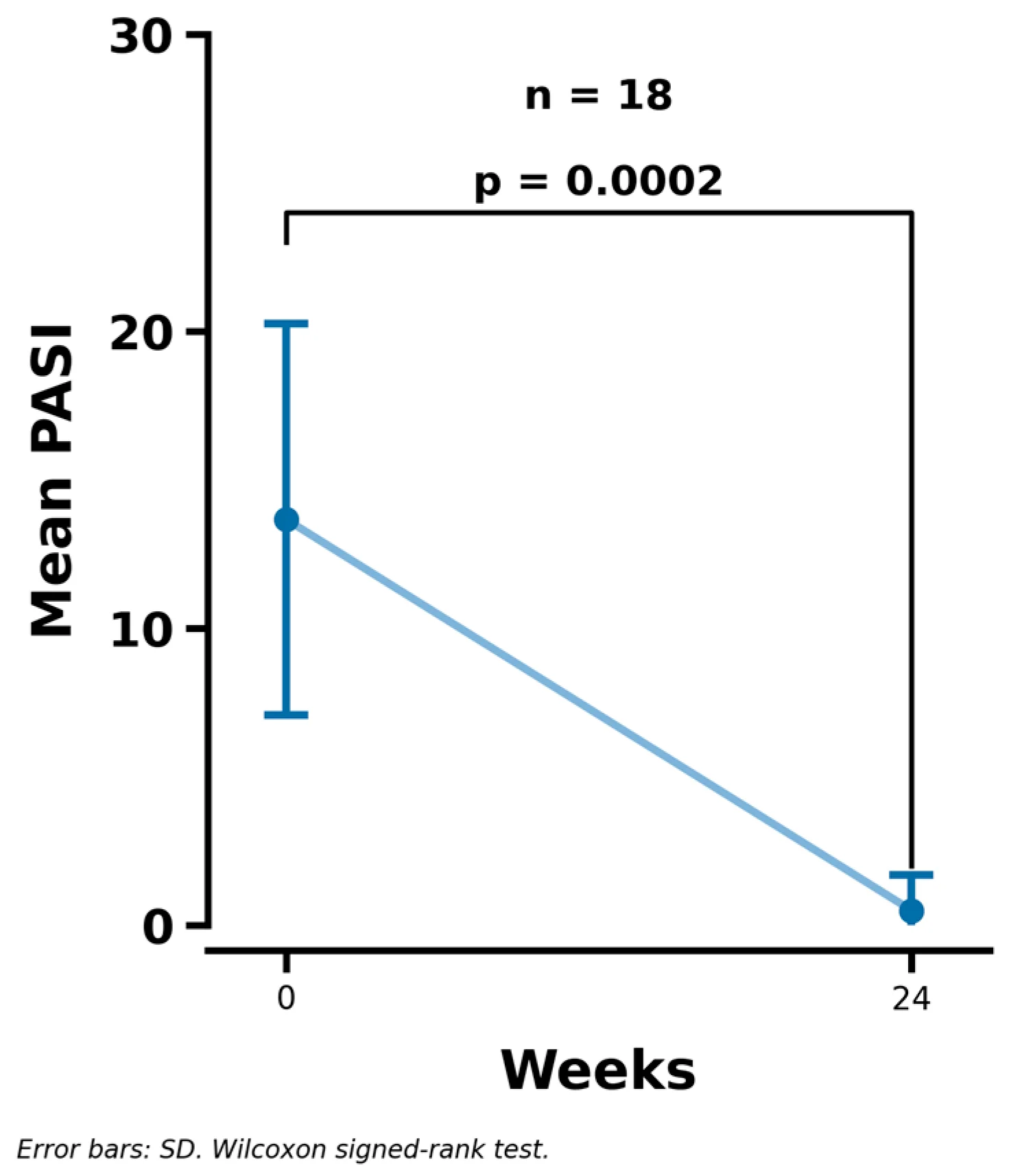
Mean Psoriasis Area and Severity Index (PASI) scores at baseline and at Week 24. A marked reduction in disease severity was observed, with mean PASI decreasing from 13.67 ± 6.59 at baseline to 0.50 ± 1.20 at Week 24, reflecting substantial improvement in cutaneous involvement following treatment. *n* = 18. Error bars represent standard deviation (SD); *p* = 0.0002, Wilcoxon signed-rank test.

Mean PASI significantly decreased from 13.67 ± 6.59 at baseline to 0.50 ± 1.20 at Week 24 (median change of −11.0; Wilcoxon signed-rank test, *p* = 0.0002) (Figure 3).

At Week 24, 18 patients (100%) achieved PASI 75, 15 patients (83.3%) achieved PASI 90, and 15 patients (83.3%) achieved complete skin clearance (PASI 100) (Figure 2).

During the 24-week observation period, no adverse events, injection-site reactions, clinically relevant laboratory abnormalities, dose adjustments, or safety-related discontinuations were documented in the available medical records.

## 4. Discussion

In this real-world cohort of heavily biologic-experienced PsA patients (100% adalimumab-exposed; 50% with multiple prior biologic failures), guselkumab Q4W produced marked improvements across joint, skin, and quality-of-life domains.

Across DISCOVER-1, DISCOVER-2, COSMOS, and APEX, guselkumab has shown consistent efficacy in both biologic-naïve and treatment-experienced patients. DISCOVER-1 demonstrated significant improvements in joint, skin, and functional outcomes, including in individuals with inadequate response to TNF inhibitors [22], whereas DISCOVER-2 confirmed substantial clinical efficacy in biologic-naïve patients, with both dosing regimens achieving markedly higher ACR20 responses versus placebo. Notably, in DISCOVER-2, only the Q4W regimen significantly inhibited radiographic disease progression at Week 24 compared to placebo, supporting its European approval for patients at high risk of joint damage [18].

The favorable outcomes observed in our cohort should be interpreted considering both statistical and methodological factors. Regarding cutaneous response, the 100% PASI75 rate observed at Week 24 (95% CI: 82.4–100%, reflecting the limited sample size) is statistically compatible with the 86.5% PASI75 rate reported in the guselkumab q4w arm of DISCOVER-1 [22]. Importantly, the favorable efficacy observed in our biologic-experienced cohort is consistent with DISCOVER-1, in which guselkumab demonstrated robust efficacy irrespective of prior TNF inhibitor exposure, including in patients with inadequate response to one or two TNF inhibitors [22]. Conversely, the higher MDA rate observed in our cohort (77.8%) compared with the 30.5% reported in the overall DISCOVER-1 q4w population should be interpreted in light of methodological differences between the two studies. Unlike DISCOVER-1, our study had an open-label, retrospective design, which may have influenced patient- and physician-reported components of MDA; no patients discontinued treatment or were lost to follow-up, thereby avoiding the non-responder imputation approach applied in the randomized trial; and treatment escalation to guselkumab Q4W was based on clinical judgment, introducing potential indication and selection bias. These methodological differences, together with the limited sample size, should be considered when interpreting the magnitude of clinical response observed in this real-world cohort.

The COSMOS study further confirmed the effectiveness of guselkumab in TNF inhibitor–inadequate responders, demonstrating meaningful improvements across both joint and skin domains [23]. Likewise, the APEX trial showed the ability of both Q4W and Q8W dosing regimens to significantly reduce bone structural damage [19]. Radiographic outcomes were not assessed in the present study, and therefore no conclusions on structural disease progression can be drawn from our data. The consistent inhibition of radiographic progression observed with the Q4W regimen in DISCOVER-2 and APEX raises the hypothesis that a similar structural benefit might be present in comparable real-world, treatment-refractory populations; however, this remains speculative and should be specifically addressed in future prospective studies incorporating imaging endpoints.

It should be noted that the clinical rationale for Q4W dosing, as reflected in its European approval, rests primarily on structural (radiographic) protection demonstrated in DISCOVER-2, a finding that APEX did not confirm as superior to Q8W in its erosion-enriched population; comparative clinical response between the two regimens has likewise not been consistently superior across trials. Accordingly, in the absence of radiographic data, our findings should not be interpreted as evidence that the Q4W regimen achieved its intended structural objective in this cohort, nor as direct evidence of a clinical advantage of Q4W over Q8W. Rather, our study represents a real-world confirmation of the clinical effectiveness and safety of guselkumab Q4W in a difficult-to-treat PsA population, marked by extensive prior biologic exposure, high baseline disease burden, and multiple comorbidities. This population—and this dosing regimen, despite its European approval—remain comparatively under-represented in the controlled trial setting. This population reflects a clinically relevant subgroup in whom current EULAR and GRAPPA recommendations support an individualized treat-to-target strategy with escalation of biologic therapy following inadequate response to previous treatment [12,13]. Dedicated prospective studies incorporating imaging endpoints are needed to determine whether this translates into the structural benefit that underlies its approved indication. Although the small sample size and retrospective data collection preclude meaningful comparison of event rates, no new safety signal emerged in our cohort. For context, adverse events of any kind were reported in 55.5% of Q4W-treated patients in DISCOVER-1 and in 38.2% of Q4W-treated patients in APEX through Week 24, with low and comparable rates of serious adverse events in both trials (0% and consistent with the overall trial population, respectively) and no new safety signals identified in either study; adverse events led to treatment discontinuation in only 0.8% of Q4W-treated patients in DISCOVER-1 [19,22]. Increased transaminases were among the most frequently reported laboratory abnormalities in the DISCOVER trials, whereas platelet count, transaminases, total cholesterol, and triglycerides remained within normal limits throughout follow-up in our cohort. While the absence of any reported adverse event or laboratory abnormality in our small, retrospective cohort cannot be formally compared to these trial-level event rates, it is consistent with the favorable and stable safety profile established for guselkumab Q4W in the pivotal literature.

### Limitations

This study has several limitations that should be considered. First, the relatively small sample size (*n* = 18) and short follow-up period (24 weeks) limit the statistical power and generalizability of our findings. Larger longitudinal studies with extended observation periods will be necessary to better define the long-term efficacy, stability of response, and safety profile of Q4W guselkumab in real-world clinical practice. Second, the retrospective, observational design is inherently subject to information and selection bias, and the absence of a valid control or comparator group precludes any causal inference regarding the relative efficacy of the Q4W schedule. Third, patients were not randomized, and the choice of dosing regimen was based on clinical judgment of risk of structural progression, which may itself have influenced outcomes (indication bias). Fourth, the study was conducted across two centers in a single Italian region, which may limit the external validity and generalizability of our findings to other populations and healthcare settings. Fifth, radiographic outcomes were not assessed, precluding any conclusion on structural disease progression, as discussed above. Finally, the cohort was heterogeneous with respect to previous biologic exposure and included two different guselkumab dosing regimens (100 mg and 200 mg every four weeks, the latter in patients with concomitant ulcerative colitis), both of which represent potential sources of confounding that should be considered when interpreting our results. Lastly, the criterion of “high risk of joint damage progression” used to select patients for Q4W dosing was applied according to clinical judgment, as no standardized operational definition is currently provided by regulatory guidance; this introduces a degree of subjectivity that should be considered when generalizing our findings to other real-world cohorts.

## 5. Conclusions

Our findings reinforce guselkumab as a reliable therapeutic option with multidomain efficacy, excellent tolerability, and a consistent safety profile. These results align with current clinical evidence supporting the role of guselkumab in controlling both articular and cutaneous inflammation [19].

Our experience is based on a limited sample size and a relatively short follow-up period, and no radiographic data were collected; therefore, this study cannot determine whether the Q4W regimen achieved its intended structural objective in this cohort, and no claim of superior therapeutic effectiveness of Q4W over the standard Q8W dosing can be made on the basis of our findings. Rather, our real-world experience indicates that guselkumab, administered as a Q4W regimen, was clinically effective and well tolerated in a heavily biologic-experienced PsA population, consistent with the established efficacy of guselkumab in this setting.

## Figures and Tables

**Table 1:** Overview of approved indications and recommended dosing regimens for guselkumab in Europe [20].

	Plaque Psoriasis	Psoriatic Arthritis	Ulcerative Colitis	Crohn’s Disease
Induction dose	100 mg SC, Wk 0 and 4	100 mg SC, Wk 0 and 4	200 mg IV, Wk 0/4/8	200 mg IV Wk 0/4/8 or 400 mg SC (2 × 200 mg) Wk 0/4/8
Maintenance dose	100 mg SC q8w	100 mg SC q8w	100 mg SC q8w, from Wk 16	100 mg SC q8w, from Wk 16
Alternative regimen	—	100 mg SC q4w—high risk of joint damage	200 mg SC q4w, from Wk 12—inadequate induction response	200 mg SC q4w, from Wk 12—inadequate induction response

Abbreviations: SC: subcutaneous; IV: intravenous; Wk: week; q4w: every 4 weeks; q8w: every 8 weeks. The alternative (Q4W) regimen is indicated for patients at high risk of joint damage progression (PsA) or with inadequate response to induction therapy (ulcerative colitis, Crohn’s disease).

**Table 2:** Summary of the demographic and clinical characteristics of the study cohort (*N* = 18).

Variable	*n*	% or SD
Sex, *n* (%)		
Male	8	44.4%
Female	10	55.6%
Age, years (mean; SD)	54.7	12.64
BMI, kg/m^2^ (mean; SD)	30.3	4.59
Smokers, *n* (%)	9	50.0%
Ex-smokers, *n* (%)	2	11.1%
PsA, *n* (%)	18	100%
Disease duration, years (mean; SD)	12.2	8.43
Baseline disease activity and inflammatory markers (mean; SD)		
DAPSA	34.3	7.6
Pain VAS, cm	5.89	1.28
DLQI	15.94	7.41
PASI	13.67	6.59
CRP, mg/dL	0.33	0.23
Articular domains		
Predominant peripheral arthritis	13	72.2%
Predominant axial disease	5	27.8%
TJC68 (mean; SD)	13.2	4.7
SJC66 (mean; SD)	7.4	3.1
Dactylitis	7	38.9%
Enthesitis	11	61.1%
Nail psoriasis, *n* (%)	10	55.6%
Prior conventional systemic therapies (multiple options), *n* (%)		
Methotrexate	16	88.9%
Cyclosporine	6	33.3%
Sulfasalazine	3	16.7%
Prior biologic therapy (multiple options), *n* (%)		
Adalimumab	18	100%
Infliximab	2	11.1%
Secukinumab	5	27.8%
Ixekizumab	2	11.1%
Comorbidities (multiple options), *n* (%)		
Hypertension	9	50.0%
Dyslipidemia	7	38.9%
Diabetes mellitus	7	38.9%
UC	2	11.1%

Abbreviations: SD: standard deviation; BMI: body mass index; PsA: psoriatic arthritis; TJC68: 68–tender joint count; SJC66: 66–swollen joint count; UC: ulcerative colitis. DAPSA: Disease Activity index for Psoriatic Arthritis; VAS: Visual Analog Scale; DLQI: Dermatology Life Quality Index; PASI: Psoriasis Area and Severity Index; CRP: C-reactive protein.

## Data Availability

The original contributions presented in this study are included in the article. Further inquiries can be directed to the corresponding author.
